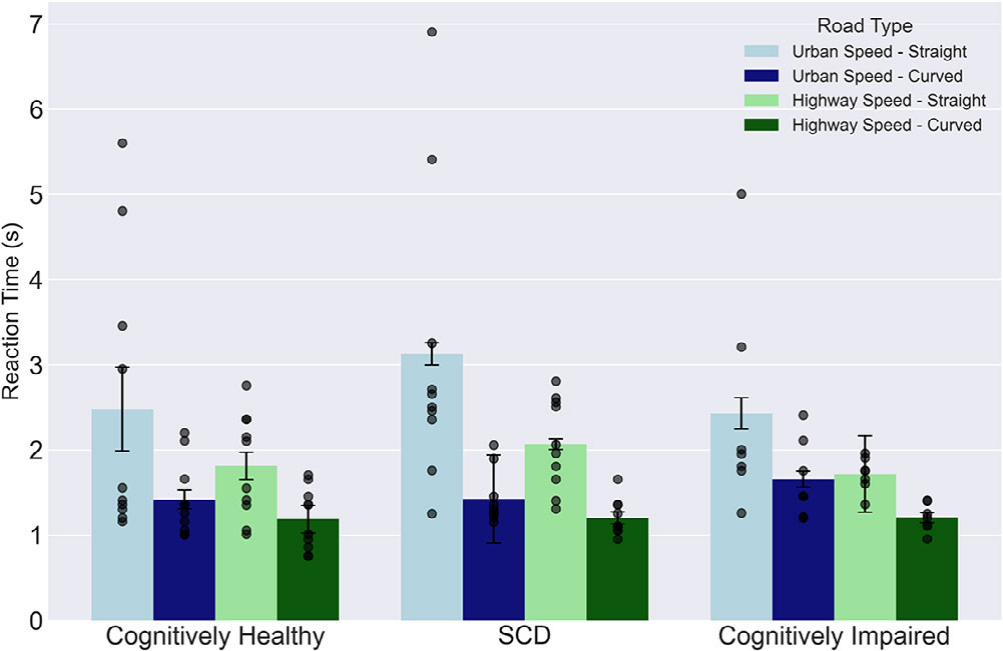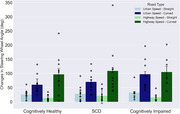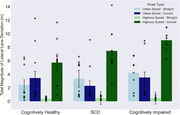# Conditionally Automated Vehicle Driving Performance Across Different Cognitive Groups

**DOI:** 10.1002/alz70858_105808

**Published:** 2025-12-25

**Authors:** Gelareh Hajian, Bing Ye, Elaine Stasiulis, Mark J. Rapoport, Gary Naglie, Alex Mihailidis, Jennifer L. Campos

**Affiliations:** ^1^ KITE ‐ Toronto Rehabilitation Institute, University Health Network, Toronto, ON, Canada; ^2^ University of Toronto, Toronto, ON, Canada; ^3^ Baycrest Health Sciences, Toronto, ON, Canada; ^4^ Sunnybrook Health Sciences Centre, Toronto, ON, Canada; ^5^ Toronto Rehabilitation Institute, Toronto, ON, Canada

## Abstract

**Background:**

Driving cessation due to cognitive decline impacts independence and quality of life. Conditionally Automated Vehicles (CAVs) may extend safe mobility for older adults with cognitive impairments by handling most driving tasks while requiring driver control in emergencies. However, the extent to which critical safety measures such as reaction time and the ability to takeover control of the vehicle during emergencies, differ across persons with different levels of cognitive impairment remains underexplored. This study addresses this gap by comparing the driving performance of older adults with normal cognition, subjective cognitive decline (SCD), and cognitive impairment (mild cognitive impairment and very mild dementia).

**Method:**

Participants included those with normal cognition (*n* = 10, age: 74.4 ± 5.36), SCD (*n* = 10, age: 75.8 ± 5.37), and cognitive impairment (Clinical Dementia Rating = 0.5, *n* = 7, age: 75.4 ± 6.45). In a high‐fidelity driving simulator, they completed four takeover requests (TORs) during a ten‐minute CAV drive, assuming control when operational limits were reached on straight and curved roads at urban and highway speeds. Takeover performance (reaction time, steering angle changes, and lane deviation) was analyzed using repeated‐measures ANOVA to assess cognitive group, road geometry, and speed effects.

**Result:**

No significant main effect of cognitive group or interactions with cognitive group on takeover performance were observed, though low statistical power may have limited detection. Road geometry influenced all groups, with faster reaction times, larger steering angle changes, and greater lane deviations on curved than straight roads. Road speed affected reaction time, with slower reactions in urban than highway sections. Although road speed did not significantly impact steering angle changes or lane deviations, its interaction with road geometry showed that curved roads at highway speeds resulted in greater steering angle changes and lane deviations than urban speeds. These findings highlight the impact of road geometry and speed on takeover performance, with high‐speed curved roads posing greater challenges for all cognitive groups.

**Conclusion:**

While takeover performance did not differ across cognitive groups, suggesting CAVs may support older adults with cognitive impairments, the small sample limits confidence. Further research with larger datasets is needed to ensure safe and effective CAV use for this population.